# Two parallel, pragmatic, UK multicentre, randomised controlled trials comparing surgical options for upper compartment (vault or uterine) pelvic organ prolapse (the VUE Study): study protocol for a randomised controlled trial

**DOI:** 10.1186/s13063-016-1576-x

**Published:** 2016-09-08

**Authors:** Cathryn Glazener, Lynda Constable, Christine Hemming, Suzanne Breeman, Andrew Elders, Kevin Cooper, Robert Freeman, Anthony R. B. Smith, Suzanne Hagen, Alison McDonald, Gladys McPherson, Isobel Montgomery, Mary Kilonzo, Dwayne Boyers, Beatriz Goulao, John Norrie

**Affiliations:** 1 Health Services Research Unit, University of Aberdeen, Foresterhill, Aberdeen, AB25 2ZD UK; 2 Centre for Healthcare Randomised Trials (CHaRT), University of Aberdeen, Aberdeen, UK; 3 Department of Obstetrics and Gynaecology, Aberdeen Royal Infirmary, NHS Grampian, Aberdeen, UK; 4 Nursing, Midwifery and Allied Health Professions Research Unit, Glasgow Caledonian University, Glasgow, UK; 5 Department of Obstetrics and Gynaecology, Plymouth Hospital NHS Trust, Plymouth, UK; 6 Warrell Unit, St Mary’s Hospital, Manchester, UK; 7 Independent consumer representative, Aberdeen, UK; 8 Health Economics Research Unit, University of Aberdeen, Aberdeen, UK

**Keywords:** Uterine, Vault, Prolapse, Hysterectomy, Suspension, Symptoms, Randomised controlled trial

## Abstract

**Background:**

One in three women who have a prolapse operation will go on to have another operation, though not necessarily in the same compartment. Surgery can result in greater impairment of quality of life than the original prolapse itself (such as the development of new-onset urinary incontinence, or prolapse at a different site). Anterior and posterior prolapse surgery is most common (90 % of operations), but around 43 % of women also have a uterine (34 %) or vault (9 %) procedure at the same time. There is not enough evidence from randomised controlled trials (RCTs) to guide management of vault or uterine prolapse. The Vault or Uterine prolapse surgery Evaluation (VUE) study aims to assess the surgical management of upper compartment pelvic organ prolapse (POP) in terms of clinical effectiveness, cost-effectiveness and adverse events.

**Methods/design:**

VUE is two parallel, pragmatic, UK multicentre, RCTs (Uterine Trial and Vault Trial). Eligible for inclusion are women with vault or uterine prolapse: requiring a surgical procedure, suitable for randomisation and willing to be randomised. Randomisation will be computer-allocated separately for each trial, minimised on: requiring concomitant anterior and/or posterior POP surgery or not, concomitant incontinence surgery or not, age (under 60 years or 60 years and older) and surgeon. Participants will be randomly assigned, with equal probability to intervention or control arms in either the Uterine Trial or the Vault Trial. Uterine Trial participants will receive either a vaginal hysterectomy or a uterine preservation procedure. Vault Trial participants will receive either a vaginal sacrospinous fixation or an abdominal sacrocolpopexy. Participants will be followed up by postal questionnaires (6 months post surgery and 12 months post randomisation) and also reviewed in clinic 12 months post surgery. The primary outcome is the participant-reported Pelvic Organ Prolapse Symptom Score (POP-SS) at 12 months post randomisation.

**Discussion:**

Demonstrating the efficacy of vault and uterine prolapse surgeries is relevant not only to patients and clinicians but also to health care providers, both in the UK and globally.

**Trial registration:**

Current controlled trials ISRCTN86784244 (assigned 19 October 2012), and the first subject was randomly assigned on 1 May 2013

**Electronic supplementary material:**

The online version of this article (doi:10.1186/s13063-016-1576-x) contains supplementary material, which is available to authorized users.

## Background

The reasons for the trial (see also Additional file 1 for further background):

Gynaecologists have recognised for some time that both anatomical failure and recurrence of prolapse symptoms after surgery are common: one in three women who have a prolapse operation will go on to have another, though not necessarily in the same compartment [1]. More recently, it has also been recognised that surgery can be followed by a greater impairment of quality of life than from the original prolapse itself (e.g. the development of new-onset urinary incontinence after surgery, or prolapse at a different site). While anterior and posterior prolapse surgery is most common (90 % of operations), around 43 % of women also have a uterine (34 %) or vault (9 %) procedure at the same time. Indeed, this demonstrates that women who have a hysterectomy have around a 27 % chance of needing a subsequent vault prolapse repair.

These data are derived from the first 700 women recruited in PROSPECT (PROlapse Surgery: Pragmatic Evaluation and randomised Controlled Trials in women with anterior or posterior pelvic organ prolapse), a large HTA-funded UK randomised controlled trial (RCT) of anterior or posterior prolapse surgery with or without the use of mesh (HTA No. 07/60/18) also being undertaken by the research team. VUE is a follow-on trial in which women will be randomised to different surgical options for upper compartment prolapse (vault and uterine). This protocol sets out our plans for running VUE.

Our aim is to identify the optimal surgical procedures for women with a vault or a uterine prolapse, which will provide women with the best symptomatic cure and lowest adverse effect and reoperation rates. Given the number of prolapse procedures currently performed and the anticipated rise in the need for such surgery with an ageing population, [2] the potential cost implications for the health service are considerable. The findings will guide gynaecologists in their surgical practice and purchasers in their choice of provision of health care. The study aims to identify which procedures are not only most clinically effective but also most cost-effective.

## Summary of evidence base

Systematic reviews have demonstrated that there was not enough evidence to guide practice for women contemplating surgery for their vault or uterine prolapse [3, 4]. The trials for vault surgery were individually too small to be conclusive and, hence, guide practice. Three RCTs broadly addressed hysterectomy versus uterine preservation approaches, but differences in the approaches and techniques used, and the outcome measures reported, precluded any useful meta-analysis or conclusions. Thus, there is no reliable evidence to guide women and their gynaecologists in choosing the best surgical cure for vault or uterine prolapse.

## Implications for proposed project

There is insufficient information about any of the surgical options to guide management of vault or uterine prolapse in women. We have identified, from PROSPECT and Health Episode Statistics (HES) data that around a third of women with prolapse have a hysterectomy for uterine prolapse. At least 25 % (HES data [2]) and 27 % (PROSPECT data) of these women go on to have a subsequent vault prolapse procedure. The evidence base for treating either of these groups of women is clearly inadequate, with very little evidence regarding subjective prolapse symptoms, effect on quality of life, cost-effectiveness and safety.

This study will fulfil the research need identified by the Cochrane review [3] and the Interventional Procedures (IP) review [4] for adequately powered RCTs of the surgical options for women having vault or uterine prolapse surgery. It will comprise two of the largest, rigorous and independent RCTs comparing traditional prolapse operations with each other. We will take into account the different clinical characteristics of women having concomitant procedures such as anterior or posterior repair, or incontinence surgery, and identify confounding factors which may predict outcomes.

## Choice of comparators

### Uterine Trial

The two options for uterine prolapse concern removal or retention of the uterus. There are a number of surgical approaches to both options (vaginal, open abdominal, laparoscopic, robotic laparoscopic) and for pragmatic reasons we intend to allow surgeons to choose the techniques which they routinely use, within certain broad categories. This will allow the maximum number of gynaecologists to recruit to VUE.

### Vault Trial

The two broad approaches to vault suspension are vaginal or abdominal. There is a general clinical opinion that the vaginal approach has a higher recurrence rate but it is simpler to perform and has less morbidity, but there is no good evidence to underpin this. Again there are different techniques to both approaches, and we intend to allow gynaecologists to use their preferred operations, again within certain broad categories.

These comparators will be further debated within the clinical and research community through the Research Committee of the British Society of Urogynaecology (BSUG) with the aim that VUE will be adopted by the Society.

## Principal objectives

The two parallel trials will compare:

- *Uterine Trial*: in women having uterine prolapse surgery, the effects of removal of the uterus versus uterine preservation
- *Vault Trial*: in women having vault prolapse surgery, the effects of a vaginal vault suspension versus an abdominal vault suspension

The primary objective is to determine the optimal surgical management for women with upper compartment (vault or uterine) pelvic organ prolapse in terms of clinical effectiveness, cost-effectiveness and adverse events (AEs).

## Secondary objectives

1. To determine the differential effects on other outcomes such as urinary, sexual and bowel function, quality of life, general health, need for secondary surgery and adverse effects
2. To identify possible effect modifiers (e.g. concomitant procedures, age, complex prolapse types)

## Methods/design

**Fig. 1:**
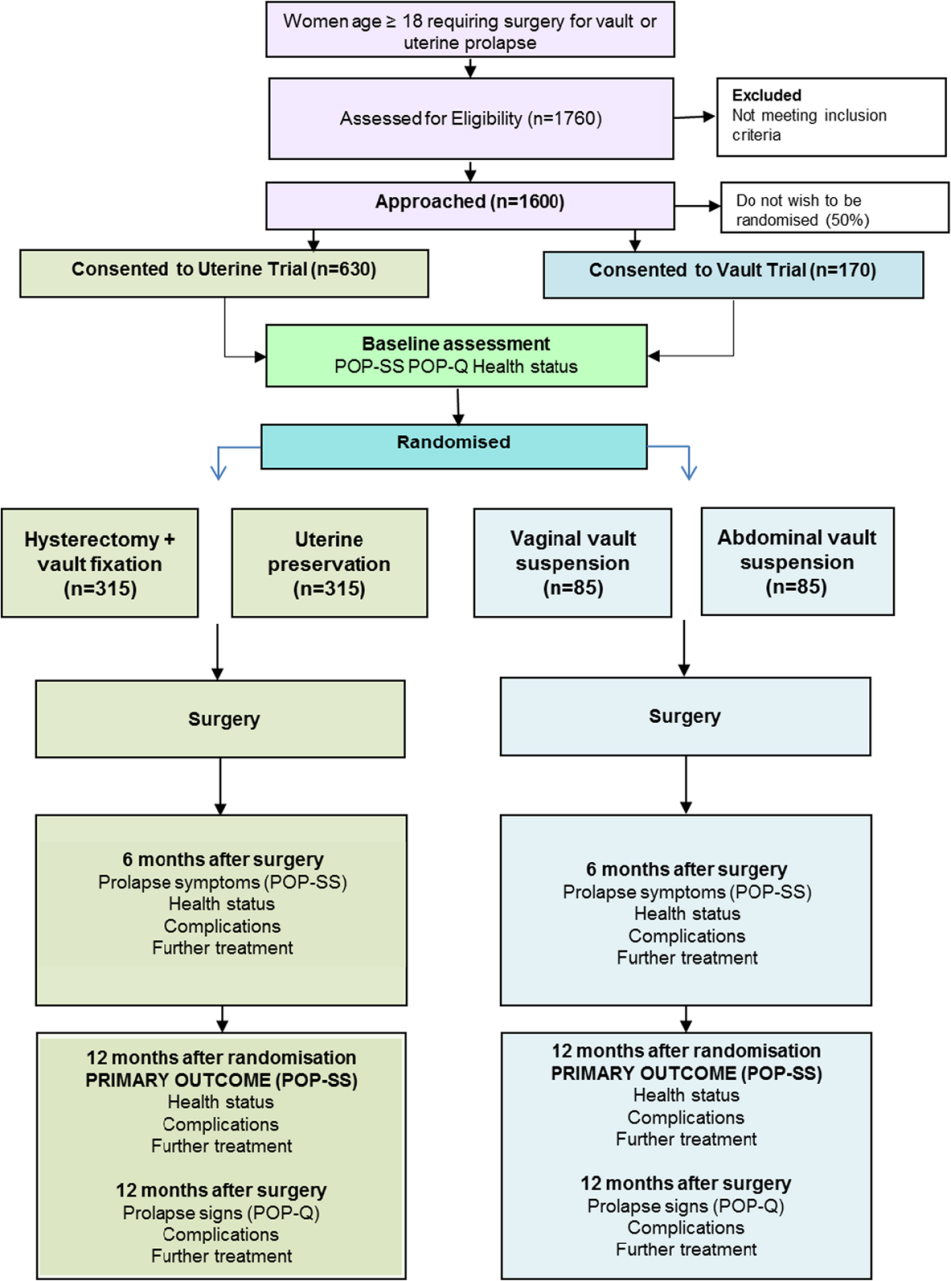
Flow diagram of study design and schedule. POP-SS (Pelvic Organ Prolapse Symptom Score), POP-Q (Pelvic Organ Prolapse Quantification)

VUE comprises two parallel, multicentre, RCTs of surgery for women with vault or uterine prolapse. The trial flow diagram is presented in Fig. 1.

### Women considered for trial entry

The study will involve women who are having prolapse surgery for vault or uterine prolapse. Two parallel but separate trials will be conducted.

A. *Uterine Trial* for women with a uterine prolapse:
  Experimental: uterine preservation (vaginal sacrospinous fixation of uterus with sutures or mesh, *or* open abdominal or laparoscopic sacrohysteropexy with a mesh bridge).
  Control (standard): vaginal hysterectomy, with a prespecified vault suspension technique using sutures or mesh if necessary
B. *Vault Trial* for women with a vault prolapse;
  Control (standard): vaginal sacrospinous fixation (with sutures or mesh or a mesh kit).
  Experimental: abdominal sacrocolpopexy (open abdominal or laparoscopic, with a mesh bridge)

Permitted concomitant procedures are:

1. Anterior and/or posterior repair,
2. Enterocele repair and
3. Continence procedure (e.g. mid-urethral sling, colposuspension)

The operations selected for VUE are described in more detail in Additional file 1.

Divergence from prespecified choices will be documented with reasons. All other operative variables will be described using standardised data collection forms. The exact surgical options will be agreed and standardised by consensus with the Research Committee of BSUG before the trial begins.

### Problems with compliance

We feel that the noncompliance rate amongst gynaecologists will be low because we will recruit gynaecologists who are uncertain about which operation to choose for their patients, and who are motivated to help with research to establish the answer.

We found that the women recruited to PROSPECT were particularly well-motivated to participate in the study: only 8.1 % declined to be recruited, 86.9 % have attended for outpatient review and there is, so far, a 94.9 % response rate to the primary outcome at 12 months.

Inclusion criteria:

- Women with vault or uterine prolapse requiring a surgical procedure
- Women who are suitable for randomisation (gynaecologist’s view, i.e. not meeting exclusion criteria)
- Women who are willing to be randomised (woman’s view)

Exclusion criteria (women who are unwilling, unable or unsuitable to be randomised):

- Previous subtotal hysterectomy
- Previous cervical amputation (Manchester repair)
- Potential future pregnancies
- Comorbidity necessitating particular approach (e.g. fibroids, previous abdominal surgery (scarring/adhesions))
- Comorbidity precluding randomisation (e.g. poor anaesthetic risk)
- Obesity precluding abdominal approach (except if two vaginal approaches are feasible)
- Colpocleisis (vaginal closure operation)

### Identification and enrolment of potential participants

All women listed for vault or uterine prolapse surgery will be identified by their treating gynaecologist and a dedicated research nurse in each centre over the 2-year recruitment period. A log will be maintained of all women meeting the eligibility criterion (listed for vault or uterine prolapse surgery), describing reasons if they do not enter the study. Every woman will be allocated a unique Study Number.

Local procedures at the participating hospitals are different and the timing and mode of approach to women and the consent process will vary to accommodate both the variability at the sites and the needs of the women. Each eligible woman will be given or sent a Patient Information Leaflet describing the study, a customised Surgical Information Sheet, a baseline questionnaire and a Consent Form. Each woman will have the opportunity to discuss the study with her gynaecologist. Women will also have the opportunity to discuss all aspects of the study with the local clinical team (staff at preadmission clinics and ward staff while admitted), the research nurse, family and friends and, if appropriate, with their GP before admission. Women may make a decision to participate during a consultation with their gynaecologist, during a visit to hospital (e.g. when they attend a clinic appointment, when they attend for preassessment or when they are admitted for surgery), or alternatively at home. If the woman agrees to be contacted at home (recorded on the Surgical Assessment Form), she may receive a telephone call from the local research nurse to discuss any queries. Women who decide to participate following telephone counselling can either send their completed documents (Consent Form and baseline questionnaire) through the post to the local team at their treating hospital or bring it with them if they are returning to hospital for another consultation or surgery.

Each woman will be asked for her signed informed consent to be randomised and followed up after her prolapse surgery by postal questionnaires and an examination in the Gynaecology Outpatient Department. The Patient Information Leaflet and the Consent Form will all refer to the possibility of long-term follow-up and being contacted about other prolapse research if the women are willing.

Each GP will be informed about each of their patients who participate in the study. Hospital staff will be informed about the study by the principal investigator (PI) and the research nurse so that they can answer queries from participants and their relatives.

All women who enter the study will be assigned a Study Number, complete a baseline questionnaire including measurement of prolapse, bowel, urinary, vaginal and sexual symptoms, and have an objective prolapse assessment (Pelvic Organ Prolapse Quantification (POP-Q) [5]) carried out by the gynaecologist or the research nurse. Women who consent will be randomised into one of the trial arms appropriate for her type of prolapse. Randomisation will be carried out as close to the time of surgery as is practical, taking into account the hospital routines and timely need for setting up the operating theatre.

All consenting women will be asked to complete the follow-up questionnaires at 6 and 12 months at home. They will also have a review appointment with their gynaecologist at 12 months to evaluate the results of surgery using the POP-Q examination and identify problems or need for other treatment. This amounts to considerably more postoperative surveillance than is available routinely in the NHS, and should ensure that all women receive optimum care.

### Number of centres involved

We aim to recruit women from approximately 40 centres, many of which also participated in PROSPECT.

### Methods to protect against sources of bias

#### Randomisation (avoiding selection bias)

When contact details, essential baseline information and confirmation of signed consent are entered into the Internet-based VUE database, the local researcher will be able to randomise the woman in the trial for which she is eligible (see flow diagram, Fig. 1). Randomisation will be carried out as close to the time of surgery as is practical taking into account the hospital routines and time needed for setting up the operating theatre.

Every woman will be logged with the Study Office and given a unique Study Number. Randomisation will utilise the existing proven remote automated computer randomisation application at the study administrative centre in the Centre for Healthcare Randomised Trials (CHaRT, a fully registered UK CRC Clinical Trials Unit) in the Health Services Research Unit, University of Aberdeen. This randomisation application will be available as an Internet-based service.

Randomisation will be computer-allocated separately for each trial and minimised on:

- need for a concomitant anterior and/or posterior POP operation or none
- need for a concomitant incontinence procedure (e.g. tension-free vaginal tape (TVT)) or not
- age (under 60 years or 60 and older), and
- surgeon

The choice of operations available to randomised women will be determined by whether they are having vault or uterine prolapse surgery. The basic criterion for joining the study is that the gynaecologist must be uncertain regarding the best operative technique for the majority of patients, and they must be competent to perform the operations to be compared (i.e. be ‘beyond’ the learning curve).

### Ensuring standardisation of intervention and outcome measurement (avoiding performance bias)

Both specialist urogynaecologists and general gynaecologists will be eligible for recruiting and randomising women, thus extending the generalisability of the trial and the future transfer of skills. All gynaecologists will be proficient in performing the POP-Q [5] method of objective quantification of prolapse descent used pre and postoperatively.

All gynaecologists will complete a Surgical Standardisation Form to provide details of their preferred operative techniques. The gynaecological surgeons who have agreed to participate in the study have extensive experience and training in prolapse surgery. Any additional training required will be conducted by the clinical grant applicants and will be directed towards ensuring standardisation of their existing techniques and outcome measures.

The research nurses and the surgeons will complete a Recruitment Officer Case Report Form (ROCRF) (developed for PROSPECT and to be adapted for VUE) at the time of surgery to ensure a complete record of all surgical techniques and materials used and any intraoperative difficulties or complications. The research nurses in each centre will ensure completeness and accuracy of data entry using remote data capture via a study web-based portal at the Study Office in Aberdeen, authored and managed by the UK CRC-registered Clinical Trials Unit in Aberdeen (CHaRT).

As this is a pragmatic trial, postoperative care will be according to local centre practice but clinical and resource-use data will be recorded.

### Loss to follow-up (attrition bias)

We have used a conservative estimate of 15 % loss to follow-up in the power calculations. We will take very active measures to minimise such loss, such as telephoning the women, using retention incentives and checks with their GPs. In addition we will obtain consent from the women to enable us to access centrally-held NHS data, for example, via the NHS Strategic Tracing Service in England and Wales, and using Community Health Index (CHI) numbers from the Information Services Division in Scotland. We have extensive experience of using such strategies and measures and have received ethics approval to do so in previous studies.

### Other sources of bias (detection bias)

Group allocation will be concealed from the woman and the ward staff if clinically possible, although blinding in theatre is not possible given that this is a surgical trial. However, we do not feel that it is necessary or ethical to perform sham incisions to conceal the route of surgery. Outcome assessment is largely by participant self-completed questionnaire, so avoiding interviewer bias. However, the clinical review at 12 months in the Outpatients Department (secondary outcome) will be conducted by staff blinded to allocation by performing the POP-Q examination, without knowledge of the actual procedure performed when possible. Participants will undergo an objective vaginal POP-Q assessment before the group allocation is revealed. Women and research staff will not be explicitly informed of which operation was randomly selected, although examination may reveal which operation was actually carried out, and women will be told if they wish to know.

Research staff who are blinded to allocation will conduct the data collection, data entry and analysis using Study Numbers only to identify women and questionnaires. All women will be actively followed up, with analysis based on the intention-to-treat principle. All analyses will be clearly predefined to avoid bias, and will be rehearsed in PROSPECT to ensure compatibility of results.

### Original sample size

In the Uterine Trial, 268 women in each arm would be required to achieve 90 % power to detect a difference in the primary outcome measure (i.e. POP-SS [6, 7] at 1 year following surgery) of 0.28 of a standard deviation at a significance level of 5 % (two-sided alpha). Allowing for 15 % loss to follow-up at 1 year would require 315 to be recruited to each arm (630 in total). The accumulating PROSPECT data indicate that a conservative estimate of the standard deviation of the primary outcome is 7 units and a difference in means of 2 units would represent a clinically important difference in POP-SS [6, 7]. Therefore, a standardised effect size of 2/7 = 0.28 standard deviations (SDs) is used.

A smaller number of women would be expected to be recruited to the Vault Trial. Using data from the women recruited to PROSPECT to date with vault or uterine prolapse, the expected number of recruits to the Vault Trial can be estimated at 27 % of that recruited to the Uterine Trial. Therefore, in the time that 630 women could be recruited to the Uterine Trial, an expected 85 women would be recruited to each arm of the Vault Trial (170 in total). A trial of 170 would have 80 % power to detect a difference of 0.43 SDs at a 5 % significance level (two-sided alpha). A standardised effect size of 0.43 equates to a difference in means of 3 units in the POP-SS measure.

In total, based on these assumptions, the number of recruits required across both trials would be 800 women.

PROSPECT data show that the mean monthly number of women randomised is 2.2 per centre and that 39 % of anterior/posterior prolapses, 259/(405 + 259) (Additional file 1) required concomitant upper vaginal prolapse surgery. Given that PROSPECT has the same inclusion criteria as this study and assuming that randomisation rates will be the same as in PROSPECT, a centre can be expected to recruit 0.86 women per month from those who would previously have been randomised in PROSPECT. PROSPECT data also show that 7 % of women presenting with vaginal prolapse require upper compartment surgery only – these women are not eligible for randomisation in PROSPECT, but would be eligible to be randomised in VUE. Assuming that randomisation rates are the same in this group of women, the expected monthly recruitment can, therefore, be inflated to 0.93 per site, i.e. 0.73 for the Uterine Trial and 0.2 for the Vault Trial. In order to recruit 630 women to the Uterine Trial and 170 to the Vault Trial, 40 centres will need to be recruited over twenty-four calendar months, allowing for a staggered start. The trial inception will be in the three centres led by the coapplicants, with recruitment subsequently rolling out to the other centres.

### Recruitment extension and increase in Vault Trial sample size

At steady state, recruitment rate of the Uterine Trial was assumed to be approximately 29 women per month. Recruitment has been slower than anticipated, and is currently averaging 15 per month. Reasons for lower recruitment include women’s preference (particularly for a hysterectomy) and lower than anticipated consent rates (around 30 % of those approached to participate in the Uterine Trial consent to do so, compared to the 50 % anticipated). As a result, an extension to the recruitment phase (an additional 15 months) is necessary to achieve the original target sample size (630).

PROSPECT data showed that the number of women requiring vault repair is approximately 27 % of the number presenting with uterine prolapse. Therefore, during the original time period for randomising 630 women to the Uterine Trial, it was anticipated that a further 170 women requiring vault repair would also be randomised to the Vault Trial.

Recruitment rates to the Vault Trial are in line with original predictions. With an additional 15 months of recruitment, the Vault Trial will continue to recruit beyond the original sample size of 170.

Conservatively assuming an average of 7 women randomised per month, we project a revised total of 280 Vault Trial participants may be recruited, which is 140 per arm or 119 allowing for 15 % loss to follow-up. This would give 80 % power to detect a difference of 0.36 SDs at 5 % significance level (two-sided alpha). A standardised effect size of 0.36 SDs equates to a difference in means of 2.5 units in the primary outcome (POP-SS), considering a SD of 7. This is a smaller difference than originally calculated (i.e. 3 units, with 80 % power). This also equates to a relative reduction in the width of the confidence interval of 22 % when compared to the precision without the extension. As the POP-SS at baseline is higher in women with vault prolapse (15.2 versus 12.0 in women with a uterine prolapse, data from PROSPECT) we could reasonably expect a greater difference after surgery.

It is anticipated that 1820 eligible women will need to be screened in order to achieve the recruitment rates required (Table 1).

**Table 1:** Recruitment numbers expected

	Uterine Trial *N* = 630	Vault Trial *N* = 280
Women needed per arm (minimum)	268	140
Allowing for 15 % dropout	315	119
Total number of women	630	280
Assuming 50 % willing to enter RCT, number of women having prolapse surgery needed	1260	560
Number of operations per year per typical centre	18	5
Number of typical centres needed for approx 39 months	35	34
Allowing for a staggered start	40	40

*RCT* randomised controlled trial

### Subsequent arrangements

#### Informing key people

Following formal trial entry:

The Study Office will inform the woman’s GP (by letter enclosing information about VUE and Study Office contact details).

The local research nurse will:

1. File the hospital copy of the Consent Form in the hospital notes along with information about VUE and the POP-Q measurements
2. Inform the ward and theatre staff as appropriate of the woman’s entry to the study and details of the intervention allocation (theatre only)
3. Use the VUE Internet database to enter data regarding the participant, including data required to complete randomisation; and intraoperative and postoperative information abstracted from local medical records
4. Return all study documentation to the Study Office in Aberdeen after database entry of essential data

### Monitoring the women

Women will be contacted by telephone, post or email as appropriate. In case of nonreturn of questionnaires, or nonattendance at outpatient appointments, attempts will be made by staff at the Study Office to trace the women directly using these means or indirectly by contacting the GP.

### Notification by GPs

GPs are asked to contact the Study Office if one of the participants moves, becomes too ill to continue or dies, or any other notifiable event or possible serious adverse event (SAE) occurs. Alternatively, staff at the Study Office may contact the GP.

### Offices for National Statistics (HES data in England, ISD data in Scotland)

Consent will be sought from all women to trace their medical records and addresses from local records and centrally held computerised databases. This should facilitate long-term follow-up.

### Ethical arrangements

We believe that the proposed research does not pose any specific risks to individual participants nor does it raise any extraordinary ethical issues.

### Data collection and processing

Follow-up will continue for 12 months from the date of randomisation (Table 2). It is not part of this protocol or the current study to follow up the women beyond this time. However, consent will be sought to make this possible in the future, and long-term follow-up is planned (Additional file 2, Protocol for long-term follow-up).

**Table 2:** Schedule for assessments/data collection

Assessment	Recruitment	Intervention (surgery)	6 months (postal questionnaire)	12 months (postal questionnaire)	12 months (clinic appointment)
Assessment of eligibility criteria	X^a^				
Written informed consent	X				
Clinical status	X^a^	X^a^			X^a^
Adverse events		X^a^	X^b^	X^b^	X^a^
Prolapse symptom score (POP-SS)	X^b^		X^b^	X^b^	
Patient-reported symptoms	X^b^		X^b^	X^b^	
Hospital admissions			X^b^	X^b^	X^a^
Health-related quality of life (EQ-5D)	X^b^		X^b^	X^b^	
Health care utilisation			X^b^	X^b^	

^a^CRF; ^b^Participant questionnaire. *EQ-5D* EuroQoL five dimensions questionnaire, *POP-SS* Pelvic Organ Prolapse Score

### Proposed outcome measures

The outcomes are identical to those piloted and used successfully in PROSPECT. We are using standardised outcome instruments developed by the International Consultation on Incontinence (ICI) [8] for urinary, bowel and vaginal symptoms, and will conform to the International Continence Society (ICS) recommendations for terminology and standard techniques [9]. We have liaised with our consumer advisor (IM) to ensure that all relevant issues are covered, the patient information and survey instruments are acceptable to the women and the outcome measures relevant.

### Use of prolapse severity score as primary outcome

Traditionally, the primary outcome of prolapse surgery has been considered to be objective (physical) restoration of normal anatomy, judged usually by the surgeon performing the operation (clinician observation). This does not necessarily correlate with women’s subjective prolapse symptoms, but these have been much more difficult to measure, not least because they can be multiple, and cure of one symptom may be accompanied by the occurrence of new symptoms. However, we strongly feel that the primary endpoint must depend on the woman’s symptoms as these alone dictate whether further treatment is requested and required. Hence, as in PROSPECT the primary clinical outcome will be the subjective difference in severity of prolapse symptoms measured using the POP-SS [6, 7].

Primary outcomes

1. The primary clinical outcome is women’s prolapse symptoms measured using the Pelvic Organ Prolapse Symptom Scale (POP-SS) [6, 7], at 1 year after randomisation
2. The primary quality of life outcome is the overall effect of prolapse symptoms on everyday life
3. The primary economic outcome measure of cost-effectiveness is incremental cost per quality-adjusted life year (QALY) (QALYs based on the EuroQol five dimensions questionnaire (EQ-5D) data) [10]

Secondary outcomes

General

- immediate and late postoperative morbidity
- other adverse effects or complications
- operating time
- blood loss
- number of nights in hospital
- number of readmissions to hospital
- need for further surgery for prolapse or for urinary incontinence
- time to further surgery
- recommendation to a friend, and
- satisfaction with surgery

Prolapse outcomes

- subjective recurrence of prolapse
- subjective continuation/recurrence of prolapse symptoms
- objective residual prolapse stage (POP-Q) at original site
- the development of new (de novo) prolapse at another site, and
- need for other conservative prolapse treatment (e.g. pelvic floor muscle training (PFMT), mechanical device)

Urinary outcomes

- urinary incontinence (persistent or de novo, and types of incontinence) using the ICI questionnaires [8]
- voiding dysfunction, and
- need for alternative management for incontinence or voiding dysfunction (e.g. PFMT, mechanical devices, surgery, drugs, intermittent catheterisation)

Bowel outcomes

- constipation (persistent or de novo), and
- faecal incontinence (persistent or de novo)

Sexual function outcome

- dyspareunia/apareunia/difficulty with intercourse, and
- vaginal symptoms using the ICI-Vaginal Symptoms Questionnaire [8]

Quality of life outcome measures

- condition-specific quality of life measures, and
- general health measures (EQ-5D) [10]

Economic outcome measures

- cost and use of NHS services
- cost to the women and their families/carers
- QALYs estimated from the responses to the EQ-5D [10], and
- the incremental costs, QALYs and incremental cost per QALY derived by the economic model over a longer-term time horizon

### Adverse effects and complications

Complications related to mesh or native tissue will be recorded and coded using the IUGA/ICS Classification Systems [11, 12]

### Questionnaires and Case Report Forms (CRFs)

#### Questionnaires for participants

Women will be asked to complete a baseline questionnaire before surgery. Content will include:

1. Prolapse symptoms (POP-SS) [6, 7]
2. EQ-5D [13]
3. Urinary outcome questions (urinary symptoms and urinary leakage, effect on quality of life [10]
4. Bowel function outcome questions and effect on quality of life
5. Vaginal and sexual symptoms, effect on quality of life [10]

The follow-up questionnaire at 6 months will enquire about:

1. Prolapse symptoms (POP-SS) [6, 7]
2. EQ-5D [13]
3. Readmissions to hospital

The follow-up questionnaire at 12 months will repeat the baseline questions and in addition will enquire about:

1. Complications and adverse effects
2. Need for further treatment for prolapse, incontinence or complications
3. Health care utilisation questions (including GP consultations and hospital visits/admissions, use of other services)
4. Personal costs (pad use, catheter use, over-the-counter medication, other health care services)
5. Satisfaction with results and recommendation to a friend

### Case Report Forms (CRFs)

#### Baseline/hospital research nurse CRF

At baseline, the research nurse will complete a Case Report Form with the following content:

Preoperative

1. Contact details, GP address, telephone numbers
2. Gynaecological and obstetric history
3. Measurement of prolapse stage using POP-Q [5]
4. Planned operative details

Intraoperative

1. Intraoperative data including date of admission and operation
2. Operative procedures and theatre time
3. Catheter and vaginal pack use
4. Complications

Postoperative

1. Pain relief, laxative use, infection, haematoma, other complications
2. Return to theatre
3. Date of discharge

### 12-month clinical review assessment form

At 12 months after surgery, all women will be examined for:

1. Clinical findings (prolapse stage using POP-Q [5])
2. Complications (e.g. mesh exposure)

### Serious Adverse Event (SAE) Report Form

Serious adverse events will be recorded using a standard SAE CRF form. The SAE form will be used to record details of any SAEs (see also ‘Safety concerns’). Adverse events will be categorised using the IUGA/ICS Classification of Complications coding systems [11, 12].

### HES and ISD data

After the last woman has been recruited, we will run periodic checks for operations, diagnoses and hospital admissions with centrally collected data to supplement and validate data collected from the participants, and to set up mechanisms for long-term follow-up.

### Data processing

Research nurses will enter locally collected data in the centres. Staff in the Study Office will work closely with local research nurses to ensure that the data are as complete and accurate as possible. Follow-up questionnaires to women will be sent from and returned to the Study Office in Aberdeen. Extensive range and consistency checks will further enhance the quality of the data.

### Change of status procedures

Participants will remain on the trial unless they chose not to receive further questionnaires and/or attend clinic appointments. We will retain their data and their permission to access health care records unless consent for these activities is explicitly withdrawn.

### Analysis plans

#### Statistical analysis

A single principal analysis is anticipated at 12 months after the last woman has had her operation. The Data Monitoring Committee (DMC) will determine the frequency of confidential interim analyses, but at present these are planned on three occasions during the data collection period.

All analyses will be based on the intention-to-treat principle. All outcomes in both trials will be described with the appropriate descriptive statistics where relevant: mean and standard deviation for continuous and count outcomes, or medians and interquartile range if required for skewed data, numbers and percentages for dichotomous and categorical outcomes (e.g. subjective recurrence of prolapse).

Analysis of the primary outcome Pelvic Organ Prolapse Symptom Score (POP-SS) will estimate the mean difference (and 95 % confidence interval) between intervention and control groups at 12 months after randomisation using a general linear model that adjusts for the minimisation covariates and other important prognostic covariates, including the baseline symptom score. A similar analysis will be used to analyse the primary outcome at 6 months after surgery.

All secondary outcomes will be analysed in a similar manner but using the appropriate generalised linear model (e.g. logistic regression for dichotomous data such as subjective prolapse failure, Poisson or negative binomial regression for count data such as number of nights in hospital) or time to event methods (e.g. Cox regression on time to further surgery) where required. We will explore analysing outcomes at all time points simultaneously using, for example, generalised estimating equations or generalised linear latent and mixed models with relevant link functions.

The ways in which these data will be analysed are set out in the VUE Statistical Analysis Plan and Dummy Tabulations. All study analyses will be according to a statistical analysis plan that will be agreed in advance by the VUE Steering Committee and compatible with that rehearsed in PROSPECT.

### Planned subgroup analyses

Subgroup analyses will be carried out within the following groups:

1. Concomitant anterior and/or posterior repair or none
2. Concomitant continence procedure or not
3. Age (below 60 years or 60 years and older)

Heterogeneity of treatment effects amongst subgroups will be tested for using the appropriate subgroup by treatment group interactions [13]. Stricter levels of statistical significance (2*P* < 0.01) will be sought, reflecting the exploratory nature of these analyses.

### Methodological analyses

The responses from women and their objective clinical findings will provide a rich data source for exploration of the correlation between patient-reported and clinician-observed outcomes, and between prolapse symptoms and their effect on quality of life. This methodological research is intended to advance the controversial field of prolapse outcome measurement, and build upon our existing work in this area.

### Proposed frequency of analyses

Women will be followed up at 6 and 12 months after randomisation. They will be asked to consent to long-term follow-up although this is not to be funded by this application. A single main analysis will be performed at the end of the trial when all 12-month follow-up has been completed. An independent DMC will review confidential interim analyses of accumulating data at its discretion but at least annually.

### Economic issues

The trial will include a formal economic evaluation assessing the costs, quality of life and cost-effectiveness of the interventions compared to the perspective of the NHS and to the women and their families. Resource-use data collected will include the cost of the intervention and the use of primary and secondary NHS services by the women including referral for specialist management. Health service costs refer to those incurred directly by the NHS due to the surgery for uterine and vault prolapse and subsequent appointments and procedures. Personal costs to the women will also be investigated.

### Resource use and costs

Health care resource use will be recorded prospectively for every patient within the study. For the surgical interventions, operative details will be recorded at the time of surgery (e.g. the time the surgery takes, the time spent in recovery, grade of surgeon and assistant, grade of anaesthetist). A parallel exercise will establish resources used immediately before, during and after (i.e. in recovery) the operation, for example, other staff, consumables (surgical requisites, mesh), and capital (costs associated with using the theatre facilities, costs of using reusable equipment). Costs to the patients will be collected using a questionnaire based on one developed by the UK working party on patient costs. The use of secondary care services (e.g. length of hospital stay, outpatient appointments, readmission) will be abstracted from patient notes or questionnaires. The use of primary care services, including medications will be collected using a patient questionnaire. Unit costs/prices will be obtained using published estimates for health care services and/or interventions.

Self-purchased health care is likely to include items such as pads bought by the participant, prescription costs and over-the-counter medications. Information about these will be collected through the health care utilisation questions.

### Quality of life

A generic instrument (the EQ-5D) [10] will be used to measure health outcomes. Trial participants will be asked to complete the EQ-5D at baseline and at 6 and 12 months after their operation and randomisation, respectively. This instrument will provide the quality of life weights to compute the QALYs.

### Cost-effectiveness

Incremental cost-effectiveness ratios will be computed comparing the cost of the interventions. The difference in effectiveness will be expressed in terms of the number of patients cured and number of patients who improve. These data will be retrieved from the participant questionnaires. Incremental cost-utility ratios will be computed comparing the interventions. The difference in utility will be expressed in terms of QALYs. Where appropriate the analysis of incremental costs, effectiveness and cost-effectiveness will be based on similar statistical models as those outlined in the statistical analysis above. Similarly, and where the data allow, the identification of appropriate subgroups for analysis will be similar to that of the statistical analysis. This ‘within-trial’ analysis will include both deterministic and stochastic sensitivity analyses to explore statistical and other forms (e.g. around unit costs or the source of utility estimates) of uncertainty.

### Modelling of longer-term outcomes

While the within-study results will prove useful it is important to note that prolapse is a chronic condition and the effects of treatment on costs and outcomes may persist into the future. An economic model which considers a longer-time horizon will be developed to provide additional information for policy-makers. In the model, the findings of the trial will be extrapolated to the patient’s lifetime. The model will describe care pathways that women may follow and will include the initial surgery and any subsequent treatments. The structure of the model will be developed in collaboration with clinicians and trial collaborators. Parameter estimates for relative effectiveness up to 1 year, costs and utilities will be derived from the trial data. Data from the trial will be supplemented with data from other sources (e.g. Cochrane reviews). Estimates of mortality will be based on data from life tables. Mortality rates will be adjusted, where necessary, using relative risks of mortality after prolapse surgery. These data will be obtained from the literature. These data will be assembled systematically and will follow guidelines for good practice [14].

Outcomes in the model will be expressed in terms of an incremental cost per QALY. Parameter uncertainty will be integrated by the incorporation of probability distributions into the model and involve Monte Carlo simulation. Other forms of uncertainty such as that associated with choices made about the structure of the model, discount rate, etc., will be addressed through sensitivity analysis. The base case and sensitivity analyses will be presented as cost-effectiveness acceptability curves (CEACs). Where data allow, the model will be reestimated for the subgroups identified above for the within-trial analysis. The model will also be used to identify priorities for further research by investigating the expected value of information.

All study analyses, including the within-trial and modelling analyses, will be conducted according to an economic analysis plan that will be agreed in advance by the VUE Steering Committee.

### Recruitment rates and milestones

#### Original recruitment rates

**Fig. 2:**
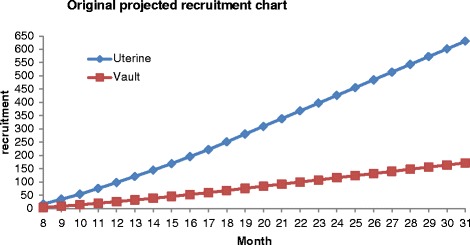
Original recruitment projection

Figure 2 shows the original projected recruitment of centres and participants, and projected number of women to be approached. Three centres will be established relatively early in the project followed by roll out to the others over the subsequent months.

The participant recruitment graph in Fig. 2 has been modelled to take into account: the phased rollout to the centres over the first 18 months; that there will be lags between the approach to women when they are in hospital for preassessment and their admission for operation; and that there are likely to be fewer operations around August and over Christmas (due to holidays).

### Revised recruitment rates with extension

**Fig. 3:**
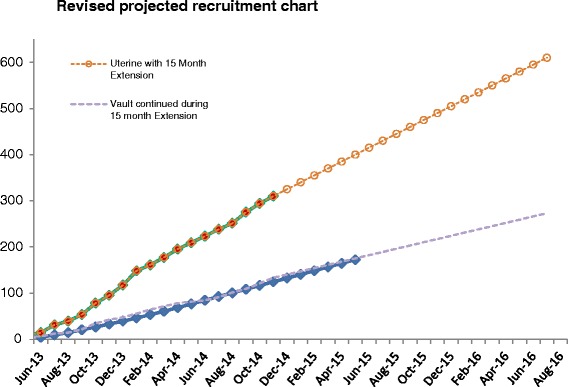
Revised recruitment projection

Due to the slower than anticipated recruitment in the Uterine Trial a 15-month extension has been approved by the TSC and DMC oversight groups and funder (February 2015). The Uterine Trial is currently averaging 15 randomised participants per month. The Vault Trial will continue to recruit (beyond the original target) during this extension. A revised recruitment graph detailing changes to both the Vault and Uterine Trials is shown in Fig. 3.

In summary, we aim to recruit 910 women to the randomised trials (630 to the Uterine Trial and 280 to the Vault Trial).

### Organisation

It is anticipated that there will be 3-monthly project management meetings, five meetings of the Steering Committee and four of the DMC. Two meetings are planned for collaborators (including gynaecologists, local research nurses, consumer participants and members of BSUG), the first is timed to occur when all the sites have been identified and the second when the results are available.

These time-related milestones will be used to enable close monitoring of progress.

### Local organisation in centres

1. Lead gynaecologist (local PI)
  Each collaborating centre will identify a lead gynaecologist who will be the point of contact for that centre. The responsibilities of this person will be to:
  establish the study locally (e.g. by obtaining agreement from clinical colleagues; facilitate local regulatory approvals; identify, appoint and train a local research nurse; and inform all relevant local staff about the study (e.g. other consultant gynaecologists, junior medical staff, secretaries, ward staff))
  take responsibility for clinical aspects of the study locally (e.g. if any particular concerns occur)
  identify which RCT the woman is eligible to participate in (vault or uterine), explain the different surgery options to them, and ensure that study documentation has been provided and that informed consent has been obtained
  notify the Study Office of any unexpected clinical events which might be related to study participation
  provide support, training and supervision for the local research nurse(s)
  represent the centre at the collaborators’ meetings
2. Local research nurse
  Each collaborating centre will appoint a local research nurse to organise the day-to-day recruitment of women to the study. The responsibilities of this person will be to:
  keep regular contact with the local lead gynaecologist, with notification of any problem or unexpected development
  maintain regular contact with the VUE Study Office
  keep local staff informed of progress in the study
  contact potential participants by: providing the Patient Information Sheet to women being admitted electively for prolapse surgery; identifying any eligible women at preassessment clinics or on the ward while they are in hospital for their prolapse surgery; explain the study and the potential for participation in a trial if they are eligible; explaining what is intended by research access to their NHS data; and describing the possibility of long-term follow-up and participation in other research
  obtain the woman’s written consent
  keep a log of whether eligible women are recruited or not (with reasons for nonparticipation)
  collect baseline data describing the women, log this information in the web-based VUE database and send paper copies to the Study Office along with the original signed Consent Forms
  use this information to randomise the women using the web-based VUE database
  ensure operative and postoperative data are collected and recorded in the web-based VUE database, and send paper copies to the Study Office
  file relevant study documentation (e.g. Consent Forms, POP-Q results) in the woman’s medical records
  organise and supervise alternative recruiters in case of holiday or absence
  represent the centre at the collaborators’ meetings

### Study coordination

#### The Study Office team

The Study Office is in CHaRT, Health Services Research Unit in Aberdeen and provides day-to-day support for the clinical centres. It is responsible for all data collection (such as mailing questionnaires), follow-up, data processing and analysis. It is also responsible for providing and maintaining the randomisation service, and communicating with the sites about VUE-specific issues. We will produce a yearly VUE newsletter for participants and collaborators to inform everyone of progress and maintain enthusiasm.

The VUE Study Office Team (Aberdeen-based grant holders and Study Office members) will meet formally at least monthly during the course of the study to ensure smooth running and trouble-shooting.

### The Project Management Group (PMG)

The study is supervised by its PMG. This consists of the grant holders and representatives from the Study Office. Observers may be invited to attend at the discretion of the PMG. We plan to meet or hold a teleconference every 3 months on average.

### The Trial Steering Committee (TSC)

The study is overseen by an independent Trial Steering Committee (TSC). The membership comprises the four independent members (including the chair), the chief investigator (CI) and grant holders. Observers or members of the sponsors (University of Aberdeen and NHS Grampian) and the funders (the HTA) may also attend, as may other members of the VUE Study Office or members of other professional bodies at the invitation of the chair. It is anticipated that the TSC will meet on five occasions.

### Research governance

The trial will be run under the auspices of CHaRT based at the Health Services Research Unit, University of Aberdeen. This will ensure compliance with research governance, and provide centralised trial administration, database support and economic and statistical analyses. CHaRT is a registered Clinical Trials Unit with particular expertise in running multicentre RCTs of complex and surgical interventions.

The CI will ensure, through the TSC, that adequate systems are in place for monitoring the quality of the study (compliance with Good Clinical Practice (GCP)) and appropriate expedited and routine reports of adverse effects, to a level appropriate to the risk assessment of the study.

### Data protection

The trial will comply with the Data Protection Act 1998 and regular checks and monitoring are in place to ensure compliance. Data are stored securely in accordance with the Act and archived to a secure data storage facility. The Consent Form will state that other researchers may wish to access (anonymised) data in the future. The senior IT manager (in collaboration with the trial statistician) will manage access rights to the data set. Prospective new users must demonstrate compliance with legal, data protection and ethical guidelines before any data are released. We anticipate that anonymised trial data will be shared with other researchers to enable international, prospective meta-analyses.

### Sponsorship

The study is cosponsored by the University of Aberdeen and NHS Grampian.

### Retention of data

It is intended to follow up the whole cohort of women for at least 10 years, and data will be retained as long as necessary for this purpose. Permissions will be sought from the relevant research governance bodies and the Ethics Committee. Attention has recently been drawn to the importance of long-term follow-up, especially in the study of pelvic floor dysfunction [15].

### Data and safety monitoring

#### Data Monitoring Committee

A separate and independent Data Monitoring Committee (DMC) will be convened. It is anticipated the members will meet once to agree terms of reference and on at least three further occasions to monitor accumulating data and oversee safety issues. This committee will be independent of the study organisers and the TSC. During the period of recruitment to the study, interim analyses will be supplied, in strict confidence, to the DMC, together with any other analyses that the committee may request. This may include analyses of data from other comparable trials. In the light of these interim analyses, the DMC will advise the Steering Committee if, in its view:

1. One of the methods of prolapse surgery has been proved, beyond reasonable doubt, to be different from the control (standard management) for all or some types of women (in respect of either effectiveness or unacceptable safety concerns)
2. The evidence on the economic outcomes is sufficient to guide a decision from health care providers regarding recommendation of which operation to choose

The TSC can then decide whether or not to modify intake to the trial. Unless this happens, however, the TSC, PMG, clinical collaborators and Study Office staff (except those who supply the confidential analyses) will remain ignorant of the interim results.

The frequency of interim analyses will depend on the judgement of the chair of the DMC. However, we anticipate that there might be two interim analyses and one final analysis.

The chair and the other independent members are to be appointed after confirmation by the HTA.

### Safety concerns

The VUE trial involves surgical operations for prolapse which are well-established in clinical practice. Adverse effects may occur after any type of prolapse surgery. The relevant guidelines for reporting SAEs will be followed.

Collaborators and participants may contact the chair of the TSC through the Study Office about any concerns they may have about the study. If concerns arise about procedures, participants or clinical or research staff (including risks to staff) these will be relayed to the chair of the DMC.

### Safety - definitions

An adverse event (AE) is defined as any untoward medical occurrence in a participant, not necessarily having a causal relationship.

All related SAEs and AEs will be recorded. Unrelated SAEs or AEs will not be recorded.

Within VUE, an SAE or AE is defined as ‘related’ if it occurs as a result of a procedure required by the protocol (i.e. prolapse surgery), whether or not this procedure is the specific intervention under investigation and whether or not it would have been administered outside the study as normal care.

Adverse events are not signs or symptoms of the disease being studied (in this case uterine or vault prolapse).

An AE is defined as ‘serious’ (SAE) if it:

- results in death
- is life-threatening
- prolongs inpatient hospitalisation*
- requires hospitalisation*
- results in persistent/significant disability/incapacity, or
- is otherwise considered medically significant by the investigator

*Hospitalisation is defined as any overnight hospital admission or day-case admission.

Adverse events which are expected after prolapse surgery are listed below. Any AEs which are deemed to be related and serious but unexpected (i.e. not on the list below) will require expedited onward reporting to the sponsor.

### Expected adverse events

In this study the following AEs are potentially expected:

Possible (expected) AEs during or associated with surgery are:

- injury to organs, blood vessels or nerves
- excess blood loss
- blood transfusion; anaesthetic complications
- death

Possible (expected) AEs following surgery are:

- excess blood loss
- haematoma
- blood transfusion
- bowel obstruction
- constipation
- faecal incontinence
- thrombosis/deep venous thrombosis/pulmonary embolism
- urinary tract infection; wound infection
- other infection (sepsis, septicaemia, abscess)
- vaginal adhesions
- pain (acute or chronic, e.g. pelvic pain/buttock pain/sciatica)
- new or persistent sexual problems including pain (dyspareunia) and inability to have intercourse (apareunia)
- perineal scarring/tightness requiring surgery (e.g. Fenton’s procedure)
- urinary retention/voiding difficulties (requiring conservative intervention, e.g. indwelling or intermittent catheterisation, drugs)
- urinary retention/voiding difficulties requiring surgical intervention (e.g. loosening/division of tape)
- new or persistent lower urinary tract symptoms (e.g. urinary incontinence, overactive bladder)
- granulation tissue (including related discharge or bleeding)
- skin tag or skin bridge
- mesh exposure/extrusion which requires no treatment or conservative treatment in clinic only (e.g. trimming, local oestrogens, silver nitrate, antibiotics)
- mesh exposure/extrusion requiring hospitalisation for surgical removal of part or all of the mesh
- other mesh complications (e.g. clumping); suture removal/trimming
- death

### Recording and reporting AEs and SAEs in VUE

#### Recording AEs in VUE

Within VUE, all related AEs and SAEs will be recorded on the Serious Adverse Event Form, CRF or participant questionnaires. In addition all deaths for any cause (related or otherwise) will be recorded on the Serious Adverse Event Form. All SAEs will be confirmed by the local PI.

### Reporting responsibilities of the CI

When the SAE form is uploaded onto the trial website, the CI or trial manager will be automatically notified. The CI (or trial manager) will report any serious and related and expected (i.e. listed above) SAEs to the sponsor within 14 days of receiving the SAE notification.

If, in the opinion of the local PI and the CI, the event is confirmed as being serious and related and unexpected (i.e. not listed above), the CI or trial manager will notify the sponsor within 24 hours of receiving the SAE notification. The sponsor will provide an assessment of the SAE. The CI (or trial manager) will report any serious and related and unexpected SAEs to the main REC and the DMC within 15 days of the CI becoming aware of it.

All related SAEs will be summarised and reported to the Ethics Committee, the funder and the TSC in their regular progress reports.

### Ethical issues and arrangements

The North of Scotland Research Ethics Committee 2 has reviewed this study. The study will be conducted according to the principles of good practice provided by Research Governance Guidelines. We believe that this study does not pose any specific risks to individual participants beyond those of any surgery, nor does it raise any extraordinary ethical issues.

### Risks and benefits

The benefit to the women participating in the trial is the chance of receiving the optimum treatment for that condition, although we do not know what that treatment is. The risks are that they may have a suboptimal operation, but any operation carries a risk and it is not known which is optimal or more risky. The benefit to women, the NHS and society is that at the end of the trial it will be known which operations are most effective and cost-effective.

### Information about risks and benefits and informed consent

Women will be informed of possible benefits and known risks of participation in the trials by means of a Patient Information Leaflet, discussion with the local research nurses and their own consultant gynaecologist. Women will be having prolapse surgery anyway, and we do not anticipate that they will run additional risks by participating in the trial. They will sign a Consent Form approved by the Ethics Committee. They will be consented to participating in the study with follow-up, being randomised, being contacted in the future about this and other research including electronic tracing using NHS data, and data linkage with computerised NHS data sources. Women who are not able or not willing to be randomised will not be recruited.

A standardised Surgical Information Sheet, similar to that in use in PROSPECT and using new Patient Information Sheets produced by the BSUG, will be adapted to provide specific clinical information for women about uterine and vault surgical options, including known complications.

### Satellite studies

It is recognised that the value of the study will be enhanced by ancillary studies of specific aspects. Plans for some of these may be submitted to other grant funding bodies. Suggestions will be discussed and agreed in advance with the TSC and also agreed with the NETSCC HTA. Appropriate legislative approvals will be sought for any new proposals.

### Indemnity

The Patient Information Leaflet provides the following statement regarding indemnity for negligent and nonnegligent harm:

‘We do not expect any harm to come to you by taking part in this study. All the materials and techniques are already being used in the NHS for prolapse surgery. Your participation in the study is therefore only to help us evaluate these procedures and should not involve any additional risk to you. Taking part in this study does not affect your normal legal rights. Whether or not you do take part, you will retain the same legal rights as any other patient in the NHS (which include professional indemnity insurance for negligence). If you wish to complain about your health care or any aspects of this study, the normal NHS mechanisms will be available to you.’

### Publication

The success of the study depends entirely on the wholehearted collaboration of a large number of women undergoing prolapse surgery, as well as their nurses and physicians. For this reason, chief credit for the study will be given, not to the committees or central organisers, but to all those who have collaborated in the study. The study’s publication policy is described in detail in Additional file 3. The results of the study will be reported first to the study collaborators. The main report will be drafted by the PMG and circulated to all clinical collaborators for comment. The final version will be agreed by the Steering Committee before submission for publication, on behalf of all the VUE collaborators.

To safeguard the integrity of the main trial, reports of explanatory or satellite studies will not be submitted for publication without prior agreement from the PMG.

We intend to maintain interest in the study by publication of VUE newsletters at intervals for participants, staff and collaborators. Once the main report has been published, a lay summary of the findings will be sent in a final VUE newsletter to all involved in the trial.

## Discussion

The VUE Study is the largest-ever randomised controlled trial on vault or uterine prolapse. The benefit to women, the NHS and society is that, at the end of the trial, it will be known which operations are most effective and cost-effective.

The main practical challenge of this trial has been participant recruitment. Early into the recruitment period we noted a patient preference for the Uterine Trial. Around 70 % of those approached declined to participate, and of these 30 % had a preference (most commonly for a hysterectomy as opposed to uterine preservation). This was somewhat expected but the extent of patient preference was higher than anticipated. The main reasons for patient preference were due to discussions with family members/friends on their experiences, GP influence and an assumption that removing the uterus (hysterectomy) would ‘cure’ the prolapse. We developed a number of strategies to improve participant recruitment, including changes to the patient information leaflet, further site training. We intend to publish this separately.

### Trial status

The first participant was randomised into the trial on 1 May 2013, and the trial is currently open to recruitment in 45 UK centres, with the last participant follow-up expected in August 2017.

VUE Protocol Version 5: 1 November 2015

## Additional files

Additional file 1: Further background information. (PDF 61 kb) Additional file 2: Protocol for long-term follow-up. (PDF 30 kb) Additional file 3: Publication policy. (PDF 22 kb) Additional file 4: SPIRIT checklist. (PDF 56 kb)

## Abbreviations

AE: Adverse event
BSUG: British Society of Urogynaecology
POP-Q: Pelvic Organ Prolapse Quantification
POP-SS: Pelvic Organ Prolapse Symptom Score
PROSPECT: PROlapse Surgery: Pragmatic Evaluation and randomised Controlled Trials
QALY: Quality-adjusted life year
