# Supplementary material for: Two parallel, pragmatic, UK multicentre, randomised controlled trials comparing surgical options for upper compartment (vault or uterine) pelvic organ prolapse (the VUE Study): study protocol for a randomised controlled trial
Source: Trials. 2016 Sep 8;17(1):441. doi: 10.1186/s13063-016-1576-x (PMC5016955; doi:10.1186/s13063-016-1576-x)
Supplement: Additional file 1: — Further background information. (PDF 61 kb) [file 13063_2016_1576_MOESM1_ESM.pdf]

ADDITIONAL FILE 1 BACKGROUND TO THE VUE STUDY

EXISTING RESEARCH

NATURE AND SCALE OF THE PROBLEM IN THE UK

The pelvic organs are normally supported by the pelvic floor (which is composed of muscle and fascia) and supporting ligaments. Pelvic organ prolapse (POP) is a herniation of the pelvic organs through a deficient pelvic floor. There is little epidemiological research into this condition because it has a variety of presentations and POP does not always cause symptoms, particularly in the early stages. [1] Estimates of the prevalence of prolapse vary from 41% [2] to 50% [3] of women over the age of 40 years.

It has been estimated that women have a lifetime risk of 11% of undergoing surgery for urinary incontinence or prolapse and 7% for prolapse alone. [4] The annual incidence of surgery for POP is within the range of 15 to 49 cases per 10,000 women years [1], and it is likely to double in the next 30 years. [5] Little is known about the prevalence and effectiveness of different types of operations, but they are notoriously prone to failure: around 30% of women undergo further operations. The mean time interval to a secondary operation is about 12 years, and the time interval between subsequent procedures decreases with each successive repair. [4] In addition, repair of one type of prolapse may predispose the women to the development of a different type of prolapse in another compartment of the vagina due to alteration in the dynamic forces within the pelvis. [4]

Pelvic organ prolapse affects a woman's quality of life by its local physical effects such as pressure, bulge or discomfort or its effect on urinary, bowel or sexual function. [6] Urinary symptoms can include frequency, urgency, urgency incontinence and incomplete emptying. Bowel symptoms are also apparent usually with a posterior

vaginal wall prolapse. Sexual symptoms include difficulty or inability to have intercourse due to pain, obstruction or embarrassment.

USE OF NHS RESOURCES

Prolapse surgery is common: in England and Wales in 2010-11, 35,668 women were admitted to hospital with a main diagnosis of female genital prolapse, and 36,043 operations were performed (some women had more than one type of prolapse operation while others had no surgery [7]). Assuming a population based on the 2010 estimate [8] of 9.3 million women in the age group at risk for prolapse surgery (50 to 85 years), the operation rate in England and Wales is currently around 382 women having prolapse operations per 100,000 per year. The need is likely to increase due to the rising number of elderly women. It has been projected that the number of women in the age group 50 to 85 years (i.e. those most likely to need prolapse surgery) will increase by 1.4m (15%) between 2012 and 2022. [8] Furthermore, growth in demand for services to care for female pelvic floor disorders will increase at twice the rate of growth of the same population. [5]

While the majority of the operations (26,673/36,043, [7] 74%) were in women having anterior and / or posterior repair, a significant proportion of women (21%) were having a uterine prolapse repair (7478). A further 1892 (5%, or 25% of the number of women having a uterine prolapse repair) were having a vault repair. These on-line HES data may underestimate total procedures, as they were based only on numbers of main operations and therefore did not count concomitant procedures.

EVIDENCE BASE FOR THE MANAGEMENT OF PROLAPSE

There is little evidence available from RCTs to guide management for women with vault or uterine prolapse. Three Cochrane reviews cover the main options: surgical management; [9][9](Maher, Feiner et al. 2010)(9)(9)[9] and conservative management, which includes: mechanical devices; [10] and physical treatment such as pelvic floor muscle training (PFMT). [11]

CONSERVATIVE MANAGEMENT FOR WOMEN WITH PROLAPSE

Although there are no RCTs to guide the use of mechanical devices (pessaries or rings), [10] these are often used for women who are unfit for surgery or who wish to avoid surgery. They can be very effective, but questions remain about the best type of device, long term adverse effects and the use of supplementary treatment such as oestrogen. Further research is required.

Conservative physical treatments such as pelvic floor muscle training (PFMT) are also often recommended as first line management. A recent update of the relevant Cochrane review [11] found some limited evidence that PFMT may reduce prolapse symptoms and severity. PFMT can also be used as an adjunct to surgery: in this situation the evidence is contradictory. However, further research is required as some of the trials were small or of poor quality.

SURGICAL MANAGEMENT FOR WOMEN WITH VAULT OR UTERINE PROLAPSE

A recent unpublished update of the Cochrane Review of surgery for prolapse [9] identified seven trials of surgical interventions for women with vault prolapse and five addressing uterine prolapse. Differences in inclusion criteria or interventions (e.g. types of women, types of operations) precluded much useful meta-analysis or reliable conclusions.

UTERINE PROLAPSE

Five RCTs compared different types of prolapse repairs for women with uterine prolapse:

- Abdominal uterine preservation versus vaginal hysterectomy and repair (n=82, Roovers 2004, 2008 [12];
- Vaginal sacrospinous uterine suspension versus vaginal hysterectomy (n=65, Dietz 2010 [13]; n=158, Jeng 2005 [14];
- Abdominal hysterectomy and sacral colpopexy versus vaginal hysterectomy and a vault support procedure (Mayo McCall) (n=47, Braun 2007 [15]);
- Hysterectomy with high levator myorrhaphy (HLM) versus hysterectomy with uterosacral vaginal vault suspension (UVVS) (n=229, Natale 2010 [16]).

The first three of these trials broadly addressed hysterectomy versus uterine preservation approaches, but differences in the approaches and techniques used, and the outcome measures reported, precluded any useful meta-analysis or conclusions. The other two trials compared two methods of vault suspension after hysterectomy to prevent later vault prolapse. Thus there is no reliable evidence to guide women and their gynaecologists in choosing the best surgical cure for uterine prolapse.

VAULT PROLAPSE

Seven small RCTs reported comparisons between a variety of different operations for vault repair. None of the trials could be combined for meta-analysis because the operations were too dissimilar.

Abdominal versus vaginal approaches:

- Abdominal sacral colpopexy versus high uterosacral ligament suspension (n=110, Rondini 2011 [17]);

- Laparoscopic sacral colpopexy versus total vaginal mesh (a mesh kit, TVM) (n=108, Maher 2011 [18]);
- Abdominal sacral colpopexy versus vaginal sacrospinous colpopexy (n=89, Maher 2004 [19]).

Abdominal versus abdominal approaches:

- Open abdominal sacral colpopexy versus laparoscopic sacral colpopexy (n=53, Pantazis 2011 [20]);
- Laparoscopic sacral colpopexy versus robotic sacral colpopexy (n=67, Paraiso 2011 [21]);
- One type of mesh bridge versus another type of mesh bridge for sacral colpopexy (n=100, Culligan 2005 [22]).

Vaginal versus vaginal approaches:

- Vaginal sacrospinous colpopexy versus posterior intravaginal slingplasty (IVS, a type of mesh kit, infracoccygeal sacropexy) (n=66, Meschia 2004 [23]).

The trials were individually too small to be conclusive and hence guide practice. Thus there is no reliable evidence to guide women and their gynaecologists in choosing the best surgical cure for vault prolapse either.

FURTHER EVIDENCE FOR SURGICAL MANAGEMENT OF VAULT AND UTERINE PROLAPSE IN WOMEN

An Interventional Procedures Review has also been conducted on the use of mesh in upper compartment prolapse. [24] The Interventional Procedures Advisory Committee (IPAC) considered the evidence from this systematic review [24] and, based on the evidence from both RCTs and non-randomised studies, produced guidance on:

- sacrocolpopexy with hysterectomy using mesh for uterine prolapse repair IPG284
- insertion of uterine suspension sling (including sacrohysteropexy) using mesh for uterine prolapse repair IPG282

- infracoccygeal sacropexy using mesh for uterine prolapse repair, IPG280
- infracoccygeal sacropexy using mesh for vaginal vault prolapse repair, IPG281
- sacrocolpopexy using mesh for vaginal vault prolapse repair, IPG283

Of these procedures, only the standard operation of sacrocolpopexy using mesh for vault prolapse (IPG283) was considered to have enough evidence for safety and efficacy, such that it could be used under normal arrangements. The other procedures 'should only be used with special arrangements for clinical governance, consent, and audit or research'.

THE OPERATIONS

SURGERY FOR VAULT AND UTERINE PROLAPSE

This application is concerned with surgical operations for vault and uterine prolapse. For each of these sites there are several alternative traditional and new surgical techniques, none of which have been properly evaluated in adequately powered multicentre RCTs. The techniques for performing vault and uterine repairs vary widely between gynaecologists according to their training and experience.

UTERINE PROLAPSE

Uterine prolapse is the descent into the vagina of the uterus from its anatomical position in the pelvis, where it is normally held in place by ligaments and the pelvic floor. These ligaments and the pelvic floor weaken with age, childbirth, obesity, chronic cough, heavy lifting and constipation.

Removal of the uterus (hysterectomy) is standard practice at the time of prolapse repair in most parts of the world, despite the fact that descent of the uterus may be a consequence, not a cause of prolapse. [25] Hysterectomy is not, however, an evidence-based practice. Women may wish to avoid hysterectomy if they wish to have more children or because they believe that the uterus is important for both their own sexuality and sexual satisfaction. Women may also wish to avoid adverse effects such as operative morbidity, damage to pelvic nerves and vessels and later incontinence. [25] Surgery for uterine prolapse is broadly divided into two approaches: hysterectomy or repair with uterine preservation.

Uterine removal (hysterectomy)

When the uterus is removed at hysterectomy, the support at the top of the vagina, the vaginal vault, must be secured. This is generally performed by:

- Vaginal hysterectomy +/- vault support procedure such as plication of uterosacral and cardinal ligaments; or
- Total abdominal hysterectomy +/- vault support procedure such as sacrocolpopexy (attachment of the vaginal vault to the sacrum with mesh).

Uterine preservation

- Amputation of the cervix with shortening and apposition of the cardinal ligaments;
- Sub-total abdominal hysterectomy (supracervical hysterectomy) and sacrocervicopexy (attaching the cervical stump to the sacrum with mesh); or
- Hysteropexy (attaching the uterus to the sacrospinous ligaments or sacrum, with sutures, mesh or a mesh kit).

Of the uterine preservation operations, we are limiting the procedures in VUE to methods of hysteropexy only.

VAULT PROLAPSE

Vault prolapse occurs in women after a hysterectomy when the vaginal vault (top of the vagina) descends into or out of the vagina. Vault support procedures carried out at the time of hysterectomy (described above) may not prevent subsequent vault prolapse. The abdominal contents (bowel) will usually descend with it, which is termed an enterocele. If the anterior vaginal wall also descends, the bladder and/or urethra descends too (cystocele, urethrocele). If the posterior wall is involved, the rectum (rectocele) or bowel (enterocele) may also descend.

For women with vault prolapse, a variety of techniques to suspend or reposition the vaginal apex are available. They are broadly divided into vaginal or abdominal approaches, and include:

Vaginal approaches:

- Vaginal sacrospinous fixation or colpopexy (attachment of the vault to the sacrospinous ligament, either bilaterally or on one side only: this is traditionally performed using sutures but mesh can also be used);
- Mesh kits can be used to suspend the vault using a vaginal approach.

Abdominal approaches:

- Abdominal sacrocolpopexy (attachment of the vault to the sacrum, with a mesh bridge: this can be an open abdominal, laparoscopic, or robotic laparoscopic procedure).

THE GYNAECOLOGISTS

For a gynaecologist to join the VUE study, he or she must be uncertain regarding the best operative technique for repairing prolapse, and hence be willing to randomise the majority of patients. All the gynaecologists must be able to perform two of the vault or two of the uterine operations, and be willing to randomise between them. Gynaecologists must consider themselves competent (beyond the learning curve) and in equipoise regarding their relative merits.

RELATIONSHIP BETWEEN VUE AND PROSPECT

The research team is currently running PROSPECT, HTA No. 07/60/18 **(**PROlapse Surgery: Pragmatic Evaluation and randomised Controlled Trials in women with anterior or posterior pelvic organ prolapse). PROSPECT is a UK-wide RCT of the use of mesh in anterior or posterior pelvic organ prolapse surgery in 34 UK centres. The

structure of that trial includes a Comprehensive Cohort to collect information about women who are not randomised, including those who are having an upper compartment procedure either concomitantly or alone. Hence we have up to date and very detailed information about the prevalence of vault and uterine surgery in the UK in our centres (Table 1).

Table 1 Analysis of surgical procedures in first 713 women recruited to PROSPECT: January 2009 to August 2011

**Women recruited to PROSPECT who had received surgery by August 2011**	**713**
Posterior/anterior prolapse procedure only	405 (57%)
Posterior/anterior with concomitant upper compartment prolapse procedure	259 (36%)
Upper compartment prolapse procedure only	49 (7%)
**Total number of women receiving an upper compartment prolapse procedure**	**308 (43%)**
**Total number of upper compartment prolapse procedures carried out**	**308**
Uterine	242 (79%)
Vault	66 (21%)

From these data, we concluded that 308/713, 43% of women in our centres were having an upper compartment procedure (242/308, 79% uterine and 66/308, 21% vault). Furthermore, these data imply that of the women who have a uterine prolapse (n=242) a further 66 (27%) will require a second, vault procedure in the future: in other words, there is a 27% failure rate after primary treatment of uterine prolapse. These data, from current UK practice in PROSPECT, are very similar to the on-line HES data (25%). [8]

It has taken two years to set up the PROSPECT centres, and this process is still ongoing. We have established a strong momentum with this multicentre study and

there is now a real, universal desire to address the outstanding questions in prolapse surgery. Whilst the establishment of so many participating centres has caused a delay in reaching our target recruitment rate, we have now (January 2012) randomised 796 women having anterior or posterior repairs, and recruited a further 831 women to the comprehensive cohort, in 34 centres. The HTA have recently agreed that we can continue to recruit for an extra 12 months, which will allow us to reach our target of 1450 women randomised within those having primary anterior or posterior repairs.

As a result we now have the opportunity to continue recruiting women to prolapse surgery trials in our centres. It is clear that there is no evidence to guide practice regarding the choice of operation for either uterine or vault prolapse. We have therefore surveyed our recruiting gynaecologists to establish what their current practice is, and who would be willing to continue to randomise women in VUE.

Preliminary results from our survey of PROSPECT centres suggest that almost all our gynaecologists would be willing to continue into VUE. In addition, gynaecologists from at least three new (non-PROSPECT) centres have indicated that they would be willing to consider joining. Some gynaecologists could not join PROSPECT because of mesh issues, but supply of mesh is not a problem in VUE. In addition, with the support of the British Society of Urogynaecology (BSUG), we plan to target other centres to supplement our own.

REFERENCES

[1] Hunskaar S, Burgio KL, Clark A, Lapitan MC, Nelson R, Sillen U, et al. Epidemiology of urinary (UI) and faecal (FI) incontinence and pelvic organ prolapse (POP). Incontinence: 3rd International Consultation on Incontinence. Paris, France: Health Publication Ltd, 2005:255-312.

[2] Hendrix SL, Clark A, Nygaard I, Aragaki A, Barnabei V and McTiernan A. Pelvic organ prolapse in the Women's Health Initative: gravity and gravidity. Am J Obstet Gynecol. 2002;186:1160-6.

[3] Swift SE. The distribution of pelvic organ support in a population of female subjects seen for routine gynecologic health care. Amer J Obstet Gynecol. 2000;183:277-85.

[4] Olsen AL, Smith VJ, Bergstrom JO, Colling JC and Clark AL. Epidemiology of surgically managed pelvic organ prolapse and urinary incontinence. Obstetrics & Gynecology. 1997;89:501-6.

[5] Luber KM, Boero S and Choe JY. The demographics of pelvic floor disorders: current observations and future projections. American Journal of Obstetrics and Gynecology. 2001;184:1496-501.

[6] Koelbl H, Nitti V, Baessler K, Salvatore S, Sultan A and Yamaguchi O. Pathophysiology of urinary incontinence, faecal incontinence and pelvic floor prolapse. Incontinence: 4th International Consultation on Incontinence. Paris, France: Health Publication Ltd; 2009.

[7] National Health Service (UK). Hospital Episode Statistics. http://www.hscic.gov.uk/hes.

[8] UK Government Statistics. Population estimates, England and Wales. 2007. http://www.ons.gov.uk/ons/index.html 2007.

[9] Maher C, Feiner B, Baessler K and Glazener C. Surgical management of pelvic organ prolapse in women (Cochrane Review). Cochrane Database of Systematic Reviews. 2010; doi: 10.1002/14651858.CD004014.pub4. 2010.

[10] Adams E, Thomson A, Maher C and Hagen S. Mechanical devices for pelvic organ prolapse in women (Cochrane Review). Cochrane Database of Systematic Reviews. 2006;Art. No. CD004010; doi: 10.1002/14651858.

[11] Hagen S and Stark D. Conservative prevention and management of pelvic organ prolapse in women (Cochrane Review). Cochrane Database of Systematic Reviews. 2011;Art. No. CD003882; doi: 10.1002/14651858.

[12] Roovers JP, Bleijenberg E, Schagen van Leeuwen J, Scholten P and van der Vaart H. Long term follow-up of a randomised controlled trial comparing abdominal and vaginal surgical correction of uterine prolapse (abstract number 88). Int Urogynecol J. 2008;19:591-2.

[13] Dietz V, van der Vaart CH, van der Graaf Y, Heintz P and Schraffordt Koops SE. One year follow-up after sacrospinous hysteropexy and vaginal hysterectomy for uterine descent: a randomised study. Int Urogynecol J. 2010;21:209-16.

[14] Jeng CJ, Yang YC, Tzeng CR, Shen J and Wang LR. Sexual functioning after vaginal hysterectomy or transvaginal sacrospinous uterine suspension for uterine prolapse: a comparison. J Reprod Med. 2005;50:669-74.

[15] Braun HF, Fernandez M, Dell'Oro A, Gonzalez F, Cuevas R and Rojas I. Prospective randomised study to compare colposacropexy and Mayo McCall technique in the correction of severe genital central prolapse (abstract number 19). Int Urogynecol J Pelvic Floor Dysfunct. 2007;18:S12.

[16] Natale F, La PC, Padoa A, Agostini M, Panei M and Cervigni M. High levator myorraphy versus uterosacral ligament suspension for vaginal vault fixation: a prospective, randomised study. Int Urogynecol J. 2010;21:515-22.

[17] Rondini C, Braun H, Alvarez J, Descouvieres C, Wenzel C and Aros S. Prospective-randomised study comparing high uterosacral vault suspension vs. abdominal sacrocolpopexy for the repair of apical defects and vaginal vault prolapse. (Abstract number 90). Neurourology and Urodynamics. 2010;29:939.

[18] Maher CF, Feiner B, Decuyper EM, Nichlos CJ, Hickey KV and O'Rourke P. Laparoscopic sacral colpopexy versus total vaginal mesh for vaginal vault prolpase: a randomised trial. Am J Obstet Gynecol. 2011;204:360.

[19] Maher CF, Qatawneh AM, Dwyer PL, Carey MP, Cornish A and Schluter PJ. Abdominal sacral colpopexy or vaginal sacrospinous colpopexy for vaginal vault prolapse: a prospective randomised study. Am J Obstet Gynecol. 2004;190:20-6.

[20] Pantazis K, Freeman R, Thomson A, Frappell J, Bombieri L and Waterfield M. Results from the LAS trial, an RCT comparing open abdominal to laparoscopic sacrocolpopexy for the treatment of post hysterectomy vault prolapse. Int Urogynecol J. 2008;19:S101-2.

[21] Paraiso MF, Jelosek JE, Frick A, Chen CC and Barber MD. Laparoscopic compared with robotic sacrocolpopexy for vaginal proplase: a randomised controlled trial. Obstet Gynecol. 2011;118:1005-13.

[22] Culligan PJ, Blackwell L, Goldsmith LJ, Graham CA, Rogers A and Heit MH. A randomised controlled trial comparing fascia lata and synthetic mesh for sacral colpopexy. Obstet Gynecol. 2005;106:29-37.

[23] Meschia M, Gattei U, Pifarotti P, Spennacchio M, Longatti D and Barbacini P. Randomised comparison between infracoccygeal sacropexy (posterior IVS) and sacrospinous fixation in the management of vault prolapse (abstract). Proceedings of the Joint International Continence Society (ICS) (34th Annual Meeting) and the International UroGynecological Association (IUGA), 2004.

[24] Jia X, Glazener C, Mowatt G, Jenkinson D, Fraser C and Burr J. Systematic review of the efficacy and safety using mesh or grafts in surgery for uterine or vaginal vault prolapse. 2008. http://guidance.nice.org.uk/IP284/Scope/pdf/English 2008.

[25] Brubaker L, Glazener C, Jacquetin B, Maher C, Melgrem A, Norton P, et al. Surgery for pelvic organ prolapse. Incontinence: 4th International Consultation on Incontinence. Paris, France: Health Publication Ltd, 2009:1273-320.
