# Supplementary material for: Two parallel, pragmatic, UK multicentre, randomised controlled trials comparing surgical options for upper compartment (vault or uterine) pelvic organ prolapse (the VUE Study): study protocol for a randomised controlled trial
Source: Trials. 2016 Sep 8;17(1):441. doi: 10.1186/s13063-016-1576-x (PMC5016955; doi:10.1186/s13063-016-1576-x)
Supplement: Additional file 2: — Protocol for long-term follow-up. (PDF 30 kb) [file 13063_2016_1576_MOESM2_ESM.pdf]

ADDITIONAL FILE 2 PROTOCOL FOR VUE LONG TERM FOLLOW-UP

BACKGROUND

VUE (Vault or Uterine prolapse surgery Evaluation) is two parallel randomised controlled trials to determine optimal surgical management (clinical and cost-effectiveness) of vault or uterine prolapse surgery. It is the largest trial of its kind for both vault and uterine prolapse.

The two parallel trials in VUE are evaluating, in women having prolapse surgery:

1. Uterine preservation versus hysterectomy (uterine removal) - the Uterine Trial
2. Vaginal vault suspension versus abdominal vault suspension - the Vault Trial

We aim to randomise 910 women by August 2016: 630 in the Uterine Trial and 280 in the Vault Trial. The following information is being collected in VUE:

- Demographic and surgical information at recruitment and at operation;
- Patient reported outcome data (prolapse symptoms, urinary, bowel and sexual function and EQ-5D) through questionnaires at 6 and 12 months;
- Women are examined 12 months after surgery to identify treatment success and adverse effects;
- Information about complications, readmissions, reoperations and costs.

Within the main VUE trial, short term failure (within 12 months of prolapse surgery) is captured. However, prolapse is a progressive and relapsing condition. The failure rate of prolapse surgery is high [1]: around 30% of women undergo further operations, the mean time interval to the first secondary operation is about 12 years, and the time interval between subsequent procedures decreases with each successive repair. In addition, repair of one type of prolapse may predispose the women to the development of a different type of prolapse in another compartment of the vagina due to alteration in the dynamic forces within the pelvis.

CURRENT EVIDENCE

The importance of long-term follow up has been highlighted in the literature by the gynaecology community [2] and NICE [3]. Furthermore, lack of long-term information has been highlighted by NICE as causing difficulty when counselling women for POP surgery. Long-term follow-up of all of our trial participants is essential to inform health-care commissioners, health-care professionals and consumers alike and will allow us to provide estimates of treatment effects currently not available in the literature.

METHODS

VUE participants will be contacted at 2-yearly intervals starting at 2 years from randomisation and continuing up to 12 years post randomisation. Women who did not consent to be contacted in the future for long term follow-up or who had previously withdrawn from receiving further questionnaires in VUE will be excluded from the long term follow-up study.

The questionnaires at each time point will be identical and collect the same patient-reported outcome measures currently used in VUE (and PROSPECT); prolapse symptoms; urinary, bowel and sexual function; EQ-5D; treatment failure including the need for additional surgery, pessaries, drugs or physiotherapy; postoperative complications including the need for additional surgical or conservative treatment; costs; and cost-effectiveness. In addition to postal questionnaire, HES (England) and ISD (Scotland) data will be requested to verify all preceding hospital re-admissions.

During the extended follow-up phase the same system currently used in VUE and PROSPECT will be employed to ensure questionnaire return rates are maximised. Questionnaire non-responders will be reminded by letter and further questionnaire after three weeks, followed by a reminder (postal or phone call) for continued non-responders. The death notification service

will be used to identify participants who have died. Newsletters and Christmas cards will also be used to maintain participant interest and engagement in the study.

MEASUREMENT OF COSTS AND OUTCOMES

We will use the outcomes specified in the main VUE study: the clinical primary outcome is women's report of prolapse symptoms (Pelvic Organ Prolapse Symptom Score, POP-SS [4, 5]); other outcomes include urinary, bowel and sexual function; EQ-5D; treatment failure (including further surgery for prolapse or incontinence, pessaries, drugs or physiotherapy); postoperative complications (including the need for additional surgical or conservative treatment); costs; and cost-effectiveness. Costs and benefits will be considered from the perspective of both the woman and the NHS. The primary economic outcome measure of cost-effectiveness will remain the incremental cost per QALY. The essential primary outcomes can only be captured through reports by participants via postal questionnaires. Complications, readmissions, reoperations and further treatment will be identified through a combination of 2-yearly postal questionnaires and HES and ISD data.

Analysis of the questionnaire data will be carried out and a Final Report, incorporating the data linkage results will be produced.

PRIMARY OUTCOME AND POWER CALCULATION

The primary clinical outcome for the main VUE Trial is the prolapse symptom score (POP-SS) at 12 months.

The anticipated sample sizes for VUE are 630 for the Uterine Trial and 280 for the Vault Trial. For both trials we assumed a conservative drop-out rate of 15% for the primary POP-SS at 12 months. The analysis will make use of all available information on the primary outcome within a linear mixed model with all participants contributing data at time points where it was

provided. In the Uterine Trial a best-case scenario is a drop-out of, say, 20% at 6 years which would provide approximately 90% power to detect the 12 month minimally important difference of 2 points (assuming a standard deviation of 7). A worst-case drop-out rate of 50% would give about 70% power to detect that difference. This ignores any gain in precision from serial measures on the same participants over time.

Given the reduced sample size in the Vault Trial, power will be somewhat less. Reoperation rates will accumulate over time adding precision to estimates of relative effects and providing data on likely times to these events.

The primary economic outcome measure of cost effectiveness will remain the same: the incremental cost per QALY (QALYs based on the EQ-5D [6]). In addition, because of all the potential problems associated with the use of mesh in prolapse surgery, serious adverse events will be included as a secondary outcome.

Following completion of the dataset, the full trial data will be reported for all randomised participants according to the trial Statistical Analysis Plan. Analyses will be undertaken on an intention-to-treat basis allowing for repeated (two-yearly) symptom and quality of life measurements.

MANAGEMENT

Trial management, data collection and analysis will be undertaken in the VUE Trial Office, HSRU, University of Aberdeen, under the supervised of the VUE Project Management Group. This group consists of the grant holders and representatives from the Study Office.

REFERENCES

[1] Olsen AL, Smith VJ, Bergstrom JO, Colling JC and Clark AL. Epidemiology of surgically managed pelvic organ prolapse and urinary incontinence. Obstetrics & Gynecology. 1997;89:501-6.

[2] Hilton P. Long-term follow-up studies in pelvic floor dysfunction: the Holy Grail or a realistic aim?. BJOG: an international journal of Obstetrics and Gynaecology. 2008;115:135-43.

[3] Jia X, Glazener C, Mowatt G, Jenkinson D, Fraser C and Burr J. Systematic review of the efficacy and safety using mesh or grafts in surgery for uterine or vaginal vault prolapse. 2008. http://guidance.nice.org.uk/IP284/Scope/pdf/English 2008.

[4] Hagen S, Glazener C, Sinclair L, Stark D and Bugge C. Psychometric properties of the pelvic organ prolapse symptom score. BJOG: an international journal of Obstetrics and Gynaecology. 2009;116:25-31.

[5] Hagen S, Glazener C, Cook J, Herbison P and Toozs-Hobson P. Further properties of the pelvic organ prolapse symptom score: minimally important change and test-retest reliability [abstract]. Neurourology and Urodynamics. 2010;29:1055-6.

[6] EuroQol Group. EQ-5D. http://www.euroqol.org/. Accessed January 2014.
